#  Heart Failure in the Middle East

**DOI:** 10.2174/1573403X11309020009

**Published:** 2013-05

**Authors:** Mostafa Q Al-Shamiri

**Affiliations:** Department of Cardiac Science, King Fahad Cardiac Center, King Khalid University Hospital, King Saud University, Kingdom of Saudi Arabia

**Keywords:** Heart failure, risk factors, Middle East, review.

## Abstract

The clinical syndrome of heart failure is the final pathway for a myriad of diseases that affect the heart, and is a leading and growing cause of morbidity and mortality worldwide. Evidence-based guidelines have provided clinicians with valuable data for better applying diagnostic and therapeutic tools, particularly the overwhelming new imaging technology and other, often expensive, therapies and devices, in heart failure patients. In the Middle East, progress has recently been made with the development of regional and multi-centre registries to evaluate the quality of care for patients with heart failure. A new heart function clinic recently began operation and has clearly resulted in a reduced readmission rate for heart failure patients. Many Middle Eastern countries have observed increases in the prevalence of the risk factors for the development of heart failure, including diabetes mellitus, obesity, and hypertension, with heart failure in the Middle Eastern population developing earlier than it is in their Western counterparts by at least 10 years. The earlier onset of disease is the result of the earlier onset of coronary artery disease, highlighting the need for Middle Eastern countries to establish prevention programs across all age groups. The health systems across the Middle East need to be modified in order to provide improved evidence-based medical care. Existing registries also need to be expanded to include long-term survey data, and additional funding for heart failure research is warranted.

## INTRODUCTION 

Heart failure (HF) may be defined as an abnormality in cardiac structure or function that leads to the failure of the heart to deliver oxygen at a rate commensurate with the requirements of the metabolizing tissues, despite normal filling pressures (or only at the expense of increased filling pressures) [1]. Clinically, it may be defined as a syndrome in which patients have typical symptoms (e.g., breathlessness, ankle swelling, and fatigue) and signs (e.g., elevated jugular venous pressure, pulmonary crackles, and displaced apex beat), resulting from an abnormality in cardiac structure or function [2]. Not all patients with this condition have poorly contracting ventricles and low ejection fractions. Indeed, many have uncorrected valvular disease, such as aortic stenosis or mitral regurgitation, or abnormal filling that results in diastolic HF. HF may arise from diverse causes, including hypertension, coronary artery disease, and contributing conditions, such as diabetes mellitus and obesity. HF is an increasing, global epidemic that results in significant health care expenditure, disability, and mortality [3].

In developed countries, the prevalence of HF is approximately 1%*-*2% of the adult population, with the prevalence rising to ≥10% among persons 70 years of age or older [4]. In Europe, 1 million hospitalizations are attributed to acute episodes of HF each year [5, 6]. The annual cost associated with HF in the USA is estimated to be $37 billion, due to age-related increases in prevalence and readmission rates, despite advances in medical care [7]. Examining all of the influences impacting the epidemiology of HF, the numbers of new cases of HF are likely to rise over the next few years, even if the incidence falls, chiefly because of the rapid expansion of the elderly population [8].

Comprehensive studies evaluating the prevalence of HF, and associated mortality, are lacking in the Middle East; however, scattered data from individual regions are available, including a recent Saudi prospective registry trial that reported the overall 30-day mortality rate for 1090 acute HF patients to be 7.5% [9]. The prevalence of HF in Oman is 5.17 per 1000 individuals [10]. This value appears to be less than that reported in more developed countries, possibly because of the bias in the Omani study resulting from the analysis of data from a single centre.

Over the last 10*-*15 years, there have been important advances in the diagnosis and treatment of HF. These have included diagnostic advances, particularly in the field of diagnostic imaging, and in the recognition of new diagnostic indicators, such as serum natriuretic peptides. The host of therapeutic advances over this period has included new drugs, such as the aldosterone antagonists that function as selective sinus node blockers, and implantable devices which have helped to delay the deaths of HF patients. In addition, the development of heart function clinics has led to reduced patient readmissions. The studies of heart failure in Middle East are scanty and there are no comprehensive long term studies, however through this paper the available data will be reviewed in terms of prevalence, aetiology, pathophysiology, diagnosis, treatment and prognosis of HF in the Middle East.

## PATHOPHYSIOLOGY AND CLASSIFICATION

The New York Heart Association (NYHA) classification is traditionally used to describe the clinical symptoms of HF patients, without any consideration of the aetiology or physiology of HF. The classification system is divided into 4 classes, Class I to Class IV, and is bidirectional, i.e., patients in Class I may progress to Class IV, and, with treatment, Class IV patients may improve to Class I or II. The American Heart Association guidelines, published in 2001 [11] addressed new staging for HF that emphasizes the disease’s evolution, progression, and prevention, with 4 stages being defined, A*-*D (Table **1**).

Cardiologists have borrowed a concept from cancer pathophysiology that involves the use of cytotoxic drugs to prevent stent re-stenosis by inhibiting intimal proliferation, following the use of bare metal stents for the treatment of coronary artery disease. As a result, some causes of HF are now preventable, but require screening similar to that for breast and prostate cancers [12]. Thus, a patient in NYHA Stage A may have risk factors for the development of HF, requiring his or her physician to regularly monitor those risk factors, such as hypertension, diabetes mellitus, and obesity, in order to help the patient control them. 

The emergence of different echocardiographic modalities allow further classification of HF, in the form of Heart Failure with Reduced Ejection Fraction (HF-REF) and Heart Failure with Preserved Ejection Fraction (HF-PEF). HF-PEF is defined as an ejection fraction ranging from 40 to ≤50% [9, 13]. The average numerical cut-off value for HF-PEF is ≥45%, provided that the patient does not exhibit any other signs of diastolic dysfunction, such as valvular or pericardial diseases. Based on this classification, the few Middle Eastern studies that have been conducted have revealed variable results for the prevalence of HF-PEF, 26%*-*46% (Table **2**) [9, 10, 13-17].

The diagnosis of HF-PEF is more difficult than the diagnosis of HF-REF because it is largely one of exclusion, i.e., potential non-cardiac causes of the patient’s symptoms (such as anaemia or chronic lung disease) must first be excluded (Table **3**) [18].

The differences in the prevalence of diastolic HF, or HF-PEF, in Middle Eastern studies is probably related to bias in the selection of cohorts or to the lack of accurate use of echocardiographic diagnostic criteria for diastolic dysfunction. Comparing the prevalence of HF-PEF in the Middle East to data from the USA, a similar incidence of HF-PEF is revealed (approximately 33% of HF patients) [19]. However, the Echocardiography Association of the European Society of Cardiology (ESC) has reported a higher prevalence of HF-PEF, up to 54% of HF patients, across Europe [17]. HF-PEF is more likely to be associated with older, obese, and hypertensive women, compared to HF-REF in at least one Middle Eastern study [13]. 

## AETIOLOGY OF HF PATIENTS IN THE MIDDLE EAST

Worldwide, the most common causes of HF include ischemic heart disease (IHD), hypertensive heart disease, idiopathic cardiomyopathy, and, to a lesser extent, valvular heart disease. In several Middle Eastern countries, like Oman, Saudi Arabia, and Yemen, around 52% of HF cases are secondary to IHD [9, 10, 14, 15]; in Egypt, IHD preceded HF in 66% of cases [16]. Hypertension represents the primary aetiology in 25% of HF cases in Yemen and Oman; valvular heart disease also remains a prevalent cause of HF in the Middle East, where it accounts for 22.5% of HF cases in Egypt [16], 10.5% of cases in Saudi Arabia [9], 8.4% in Oman [10], and 7% in Yemen [14]. Idiopathic dilated cardiomyopathy is responsible for a relatively small proportion of HF cases, 11% in Yemen [14] and 8.3% in Oman [10].

In the Western literature, coronary heart disease (CHD) is by far the most common cause of myocardial disease, being the initiating cause in 70% of patients with HF [20]; valve disease accounts for 10% and other cardiomyopathies for another 10% of HF cases. Compared to other Middle Eastern countries, Egypt has an exceptionally high prevalence of valvular disease. However, with the increasing prevalence of risk factors for the development of HF, the Middle East will soon be equal to Western countries when examining the prevalence of CAD as the primary aetiology for HF. This trend requires the urgent development of prevention programs for the control of these risk factors throughout the Middle East.

The prevalence of risk factors for HF is increasing. For example, 26% of the Saudi population is affected by hypertension [21] and approximately 24% is affected by diabetes mellitus [22]. Patients with diabetes are at a higher risk for developing HF; the Framingham study showed that the risk of HF was 2-fold higher among men and 5-fold higher among women with diabetes [23]. Overall, the prevalence of diabetes (both types 1 and 2) in Western patients with HF is approximately 20%*-*25% [24]. The prevalence of diabetes in HF patients, according to the Saudi registry, is 60.7% [9], which is higher than that reported by other world registries [25-31]. Age also plays an important role in the aetiology of HF, particularly in HF-PEF. In general, the Middle Eastern HF patients are 10 years younger than their counterparts in more developed countries [9, 25-31]. This is related to the increasingly high prevalence of CAD among young Arabs [32-34].

## DIAGNOSIS AND TREATMENT OF HF IN THE MIDDLE EAST

### Signs and Symptoms

The diagnosis of HF can be difficult, especially in the early stages. Although symptoms lead patients to seek medical attention, many of the symptoms of HF are non-specific and do not, therefore, help to discriminate between HF and other problems. Symptoms that are more specific (i.e., orthopnoea and paroxysmal nocturnal dyspnoea) are less common, especially in patients with milder symptoms [35-39], making them less sensitive. In the most recent ESC practice guideline, all diagnostic recommendations have been given an arbitrary evidence level of C. Class I, for diagnostic tests like complete blood counts, blood chemistry, 12-lead electrocardiograms (ECG), echocardiography, cardiac magnetic resonance imaging, right and left cardiac catheterization, and coronary angiograms. Chest radiography, natriuretic peptide level determinations, myocardial imaging, and stress ECGs were classified as Class IIc evidences. In the Euro Heart Failure survey program, the majority of patients (>90%) had had an electrocardiogram, chest radiograph, and haemoglobin and electrolytes measurements, as recommended in the ESC guidelines, but only 66% had ever had an echocardiogram. Left ventricular ejection fractions had been measured in 57% of men and 41% of women [40].

ECGs and echocardiograms are 2 of the most important diagnostic tests, and both are commonly available in the Middle East. Echocardiograms were performed in 98% of the recently conducted studies like, Heart Function Assessment Registry Trial in Saudi Arabia (HEARTS) [9], and all of the patients in an Omani study coducted in Oman had echocardiograms; ECGs and chest X-rays were also performed [10]. Echocardiograms are important for the differentiation of cardiac pathologies and the reporting of ejection fractions aids in the discrimination between systolic and diastolic types of HF. Natriuretic peptide level determinations and cardiac magnetic resonance imaging may not be available in all cardiac service centres, increasing the importance of ECGs for providing information regarding the cardiac rhythm and the presence of conduction abnormalities. The information provided by these 2 tests permit an initial working diagnosis and treatment plan for the majority of patients.

### Treatment

Overwhelming evidence, gathered from clinical trials, has proved the prognostic advantages of treating HF patients with drugs like angiotensin-converting enzymes inhibitors (ACEI), angiotensin receptor blockers (ARBS), beta-blockers, and aldosterone antagonists. A variety of clinical practice guidelines emphasize the importance of these drugs, but a considerable proportion of HF patients do not receive these medications. In a European heart survey, published in 2006 [41], 80% of patients were on ACEIs or ARBS, whereas only 61% were taking beta-blocker medications. This survey shows an obvious gap between the evidence and practice. However, a recent ESC HF survey [43] revealed satisfactory utilization of these drugs, with ACEI/ARBS being used in 86.7% of HF patients, beta-blockers in 88.5%, and aldosterone antagonists in 43.7%. This indicates that practicing physicians will change their practices over time.

In the Middle East, the use of these important medications is variable. In the HEARTS study, ACEI/ARBS use was 86%, beta-blocker use was 95%, and aldosterone antagonist use was 53%. In Assir [42, 43] (southern Saudi Arabia), a single-centre study showed that 68.3% of HF patients were on ACEI/ARBS and 51.6% were taking beta-blockers. In the Gulf countries [44], 81% of HF patients were using ACEI/ARBS and 57% were using beta-blockers.

Both cardiac resynchronization therapy (CRT) and intracardiac defibrillation (ICD) were under-used in Saudi Arabia, based upon the HEARTS report; a history of ICD and CRT insertion were documented in 29% and 8% of the patients respectively. New implant rates for ICD of 5% and CRT of 1.7% were reported.

### Prognosis 

In Europe, 50% of HF patients died within 4 years of diagnosis [45], with 13.5% of HF patients succumbing to their disease within 12 weeks of hospital admission [40], and in-hospital mortality of 6.7 % [41]. In the Middle East, in-hospital mortality was 5.3 % among acute HF patients, increasing to 7.5% at 30 days. However, this study was based in a highly equipped centre in Riyadh, the capital of Saudia Arabia [9]. A Yemeni study indicated that in-hospital mortality was 9% [15]. Another Middle Eastern study [46], reported a significant reduction in the in-hospital mortality rates over time among both men and women hospitalized with HF (1991*-*2010), with the overall mortality rate declining from 8.4% to 4.8% over the study period. This is consistent with the findings from both the Olmsted County (USA) Study (1979*-*2000) [47] and the Framingham Heart Study (1979*-*2000) [48]. 

## FUTURE DIRECTIONS

Middle Eastern countries need to develop preventive programs to combat HF due to the increased prevalence of this disease among younger people, secondary to the development of CAD. The increasing incidence of CAD among younger people is a result of an evolution towards increasingly sedentary lifestyles, combined with the increased consumption of fast food. Current services can be partly enhanced by expanding existing multi-centre registries and making provision of funds for additional research pertaining to HF and its attendant risk factors.

## Figures and Tables

**Table 1.** AHA/ACC Heart Failure Stages.

Stage	Description
**A**	People at high risk for developing heart failure, but who do not have heart failure or damage to the heart
**B**	People with damage to the heart, but who have never had symptoms of heart failure.
**C**	People with heart failure symptoms caused by damage to the heart, including shortness of breath, tiredness, inability to exercise
**D**	People who have advanced heart failure and severe symptoms difficult to manage with standard treatment

**Table 2.** Middle Eastern Studies Showing the Prevalence of HF with Reduced and Preserved Systolic Function

Country (reference)	HF-PEFa (%)	HF-REFb (%)
Saudi in-patients [9]	26.3	71.7
Saudi out-patients [13]	41*-*46	54*-*59
Egypt [16]	34.2	65.8
Oman [10]	19.9	80.1
Yemen [14,15]	30	67.8
Israel [17]	34	66

aHF-PEF, Heart Failure with Preserved Ejection Fraction; bHF-REF, Heart Failure with Reduced Ejection Fraction

**Table 3.** Criteria for the Diagnosis of HF

**The diagnosis of HF with preserved ejection fraction requires 3 conditions to be satisfied:**
1. Symptoms typical of heart failure (HF)
2. Signs typical of HF
3. Reduced left ventricle ejection fraction (LVEF)
**The diagnosis of HF with reduced ejection fraction requires 4 conditions to be satisfied **
1. Symptoms typical of HF
2. Signs typical of HF
3. Normal or only mildly reduced LVEF; left ventricle not dilated
4. Relevant structural heart disease (LV hypertrophy/left atrial enlargement and or diastolic dysfunction).
